# Clinical Application of Split-Thickness Skin with Pedicle for Finger Wounds

**DOI:** 10.1155/2018/9470198

**Published:** 2018-06-27

**Authors:** Min-Xia Zhang, Wei-Qiang Tan, Qing-Qing Fang, Chun-Ye Chen, Jian-Min Yao

**Affiliations:** ^1^Department of Plastic Surgery, The Fourth Affiliated Hospital, Zhejiang University School of Medicine, Yiwu, Zhejiang Province, China; ^2^Department of Plastic Surgery, Sir Run Run Shaw Hospital, Zhejiang University School of Medicine, Hangzhou, Zhejiang Province, China; ^3^Department of Plastic Surgery, Hangzhou Plastic Surgery Hospital, Hangzhou, Zhejiang Province, China

## Abstract

**Background:**

Skin grafts and pedicled flaps are the traditional methods of reconstructing injuries; both have some disadvantages. Here, we introduce a new clinical application of split-thickness skin with pedicle for repairing finger wounds.

**Methods:**

We present the new method of split-thickness skin with pedicle used on 12 patients (18 fingers) between 2012 and 2016. The graft was sketched on the abdomen at random according to the shape of the wounds on a skin area of 1.0 × 1.0 cm–8.0 × 1.5 cm. The pedicle was divided at 7–22 days.

**Results:**

During the follow-up time of 13–20 months, there were no reported cases of skin necrosis; 17 fingers obtained primary healing except 1, which required a dressing change.

**Conclusion:**

The split-thickness skin with pedicle proved to be valuable in the treatment of finger wounds and has the advantages of both pedicled flaps and free skin grafting.

## 1. Introduction

Finger wounds are common incidental injuries in hand surgery. Usually, the skin of the finger is lost, with exposure of tendons, bones, or joints, leading to poor hand function. Structural preservation is important as soft tissue and bone loss can result in detrimental hand disability [1]. Adequate coverage of finger wounds is urgent but challenging. Skin grafts, cross-finger flaps, and transposition flaps have been used to address the problem of soft tissue coverage [2].

Traditionally, skin grafting has been the standard method of reconstruction in finger wounds. However, this approach has many disadvantages. Its survival depends on the nutrition of the recipient site wound, which is variable. So a negative pressure in the form of suction is used under the graft and a positive pressure is applied with a bulky dressing and compression to maintain good contact with the bed to ensure a graft take [3]. The pedicled flap is more widely used to reconstruct the injury, which is safe and well established [4, 5]. However, it tends to be bulky and requires repeated procedures such as thinning, which may threaten the skin paddle vascularization.

Considering the advantages of free split-thickness skin grafting and pedicled flap, we present a new method of split-thickness skin with pedicle, using the abdominal wall to repair finger wounds between January 2010 and June 2014, with satisfactory results. The aim of this study was to introduce this new technique and evaluate whether it is valuable in finger reconstruction.

## 2. Methods

### 2.1. Patients

All patients in our study had given informed consent. Twelve patients of eighteen fingers were included in our study. Each patient presented with different finger wounds, whose causes were variable.

### 2.2. Surgical Technique

All procedures were performed under nerve blockade anesthesia combining with local anesthesia.

1. Planning in reverse was the first procedure before the surgery. The split-thickness skin with pedicle was designed on the homolateral middle or lower abdomen, and the location and direction of the pedicle were random, depending on the shape and area of the wound. The designed area of the skin graft was 1.0 × 1.0 cm–8.0 × 1.5 cm.
2. After incising three sides of the rectangular portion of the marked skin using a No. 11 scalpel blade, the remaining side became the pedicle.
3. The skin was cut, two-thirds of the dermis retained in the skin graft and one-third of the dermis in the abdominal wall were kept, then the split-thickness skin graft was raised and sutured to the finger wound.
4. The abdominal wound was sutured directly.
5. The injured hand was fixed with a bandage. The duration of the pedicle division was 7–22 days, 13 days on average, according to the time of blood circulatory blockade. If the skin graft covered in finger wounds seemed to be red and warm when the blood blocking time was longer than one hour, the pedicle can be divided safely.

## 3. Results

There were twelve patients (11 males and 1 female; 18 fingers) included in our study. Their ages ranged from 17 to 57 years (mean, 33.25 years). All patients presented with finger wounds: left hand five cases, right hand seven cases, index finger five cases, middle finger five cases, ring finger five cases, and little finger three cases. The causes of injuries were variable: hot-crush injury, 2 cases; mechanical injury, 10 cases. The duration of the pedicle division was 7–22 days, 13 days on average.

During the follow-up time of 13–20 months, there were no reported cases of skin necrosis and 17 fingers underwent primary healing except one, which required dressing changes. Two fingers got hyperpigmented, becoming lighter colored as time went on. All the skin survived well with little contraction, which was acceptable. All the patients were satisfied with their postoperative fingers, in both appearance and function and in both flexion and extension.

### 3.1. Typical Case 1

**Figure 1:**
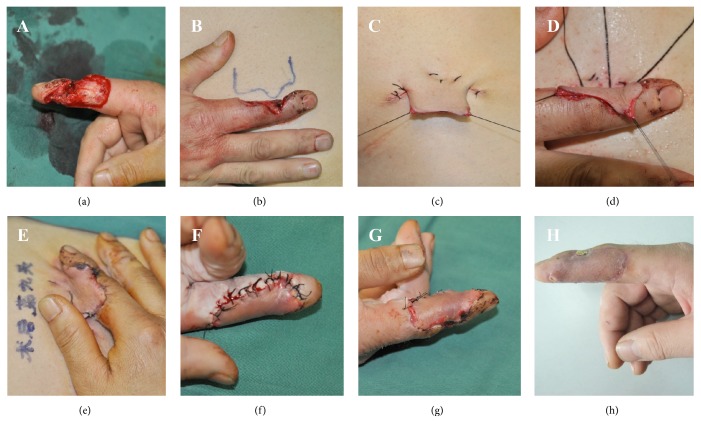
**Typical** **C** **a** **s** **e** **1**. (a) A 52-year-old man presented with a skin defect of the index finger, (b) a skin incision of 4.0 × 2.5 cm on the lower right abdomen was designed, (c) incising three sides of the skin rectangle, and cutting the split-thickness skin (two-thirds thickness), (d) raising the skin and suturing it to the finger wound, (e) the pedicle survived well on the 9th day, (f) dividing the pedicle, (g) taking half the stitches out after 7 more days, and (h) after 3 months, the skin had grown well with good appearance and nice function.

A 52-year-old man presented with an injury of the index finger of the right hand. Physical examination revealed that the skin on the radial side of the distal and middle phalanx was defective, with exposure of the flexor tendon (Figure 1(a)). We designed a skin incision of 4.0 × 2.5 cm on the lower right abdomen (Figure 1(b)), incised three sides of a skin rectangle, cut the split-thickness skin (two-thirds thickness) (Figure 1(c)), raised the skin and sutured it to the finger wound, and fixed the injured hand with a bandage (Figure 1(d)). The pedicle survived well on the 9th day (Figure 1(e)), and the time of blood circulatory blockade was longer than one hour, then we divided the pedicle (Figure 1(f)), took half the stitches out after 7 more days (Figure 1(g)), and removed the left stitches out on the 16th day. During the follow-up time, the results were satisfactory: after 3 months, the skin had grown well with good appearance and nice function (Figure 1(h)).

### 3.2. Typical Case 2

**Figure 2:**
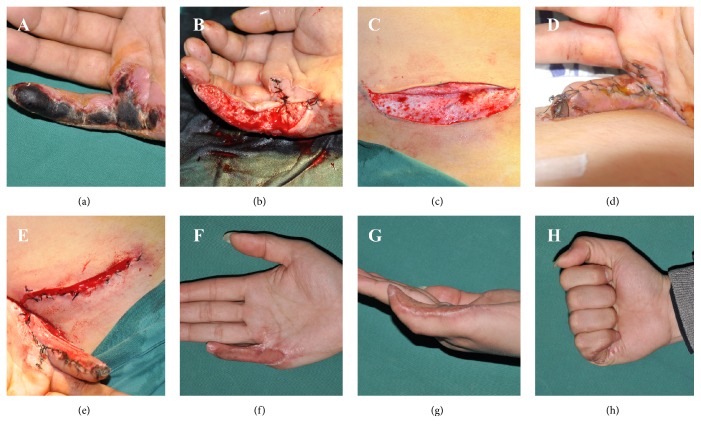
**Typical** **C** **a** **s** **e** **2**. (a) A 36-year-old man presented with a skin necrosis of the little finger, (b) excising and debriding the nonviable skin and tissue, (c) incising three sides of the designed skin incision of 8.0 × 1.5 cm, and cutting the split-thickness skin (two-thirds thickness), (d) raising the skin and suturing it to the finger wound, (e) dividing the pedicle on the 10th day, (f) the skin grew very well, although there was some light hyperpigmentation after 5 months, (g) the lamina epithelium thickened slightly, and (h) the function and activity of the finger were satisfactory.

A 36-year-old man had presented with an 8-day-old hot-crush injury of the little finger of the right hand. Physical examination showed that the skin on the ulnar side of the finger was black and necrotic (Figure 2(a)). After escharotomy and debridement of the nonviable skin and tissue in the operative site, the tendon was exposed (Figure 2(b)). We designed a skin incision of 8.0 × 1.5 cm on the ipsilateral middle abdomen, incised three sides of the skin rectangle, with the pedicle on the upper side, cut the split-thickness skin (two-thirds thickness) (Figure 2(c)), raised the skin, and sutured it to the finger wound (Figure 2(d)). The abdominal wound was sutured directly, and the injured hand was fixed with a bandage. We divided the pedicle on 10th postoperative day as the time of blood circulatory blockade was longer than one hour (Figure 2(e)) and removed all the stitches after 16 days. The patient was followed up for 5 months. The patient was satisfied that the skin of his finger grew very well, although there was some light hyperpigmentation and slight lamina epithelium thickening (Figure 2(f)). At 5 months' follow-up, function and activity of the finger were satisfactory (Figures 2(g) and 2(h)).

## 4. Discussion

Adequate coverage of finger wounds is often a challenge [6]. It lies in resurfacing the hand or finger with good quality, pliable sensate skin cover, while preserving the movements and function of the hand [3]. Traditionally, skin grafts or pedicled flaps are used, but they both have some inevitable disadvantages. With skin grafts, graft take will not be good over the exposed tendons or bones because of poor revascularization. The grafts tend to be hyperpigmented and not acceptable cosmetically. Usually, pedicled flaps offer much better reconstruction than skin grafts, but these flaps have a thick layer of subcutaneous fat, which results in a puffy and bulky shape [7]. They need secondary procedures such as flap thinning to make them more useful if necessary [8]. Another disadvantage of pedicled flaps is the long time of immobilization (at least 3 weeks). The results depend on patient compliance, which are variable. Besides patient discomfort and nursing difficulties, there is a risk of stiffening of the joints of the hand, elbow, and shoulder [9]. Here, we present a new method of split-thickness skin with pedicle to repair finger wounds, combining the merits of both skin grafts and pedicled flaps.

The artery entering into the dermis, with a diameter of 15 *μ*m, constitutes the subdermal vascular network in the reticular layer and the subpapillary capillary network in the papillary layer. There is no vessel in the epidermis [10]. In this way, the subpapillary capillary network in the papillary layer provides the blood supply for the split-thickness skin, which is two-thirds to three-fourths of the full-thickness skin. Survival of the free skin graft depends completely on the nutrition of the recipient site wound. Unlike the skin graft, the blood supply of the pedicled flap relies mostly on the pedicle. In conclusion, we brought out the split-thickness skin as a pedicle transplantation, which has a double vascular supply from the recipient site wound and the pedicle, substantially improving the survival rate. So our new technique can be used well over the exposed tendons or bones because of the double blood supply, having broader indications than free skin grafting. As well, our split-thickness skin with pedicle was thinner than flaps, with no need for secondary procedures. In addition, compared to flaps, the division time of our pedicles was shorter (usually 1 to 2 weeks) because of the excellent revascularization, which considerably reduces the risk of stiffening, patient discomfort, and nursing difficulties.

What is noteworthy is that it is important to fix the injured finger to avoid avulsion, especially under strong tension. Always be aware of the graft skin color as an indicator of viability.

The limitation of this study is its small size. Ideally, future studies will be prospective, randomized, and blinded. A longer follow-up period is needed to better characterize the effects of reconstruction.

## 5. Conclusion

The split-thickness skin with pedicle has proven to be a promising choice to cover finger wounds with exposed tendons or bones. More comparative prospective studies are needed to confirm this innovation.
